# Rac1 activation inhibits E-cadherin-mediated adherens junctions via binding to IQGAP1 in pancreatic carcinoma cells

**DOI:** 10.1186/1478-811X-7-23

**Published:** 2009-09-08

**Authors:** Beatrix Hage, Katrin Meinel, Iris Baum, Klaudia Giehl, Andre Menke

**Affiliations:** 1 Internal Medicine I, University Hospital Ulm, University of Ulm, Ulm, Germany; 2 Institute of Pharmacology and Toxicology, University Hospital Ulm, University of Ulm, Ulm, Germany

## Abstract

**Background:**

Monomeric GTPases of the Rho family control a variety of cellular functions including actin cytoskeleton organisation, cell migration and cell adhesion. Defects in these regulatory processes are involved in tumour progression and metastasis. The development of metastatic carcinoma is accompanied by deregulation of adherens junctions, which are composed of E-cadherin/β- and α-catenin complexes.

**Results:**

Here, we show that the activity of the monomeric GTPase Rac1 contributes to inhibition of E-cadherin-mediated cell-cell adhesion in pancreatic carcinoma cells. Stable expression of constitutively active Rac1(V12) reduced the amount of E-cadherin on protein level in PANC-1 pancreatic carcinoma cells, whereas expression of dominant negative Rac1(N17) resulted in an increased amount of E-cadherin. Extraction of proteins associated with the actin cytoskeleton as well as coimmunoprecipitation analyses demonstrated markedly decreased amounts of E-cadherin/catenin complexes in Rac1(V12)-expressing cells, but increased amounts of functional E-cadherin/catenin complexes in cells expressing Rac1(N17). Cell aggregation and migration assays revealed, that cells containing less E-cadherin due to expression of Rac1(V12), exhibited reduced cell-cell adhesion and increased cell motility. The Rac/Cdc42 effector protein IQGAP1 has been implicated in regulating cell-cell adhesion. Coimmunoprecipitation studies showed a decrease in the association between IQGAP1 and β-catenin in Rac1(V12)-expressing PANC-1 cells and an association of IQGAP1 with Rac1(V12). Elevated association of IQGAP1 with the E-cadherin adhesion complex via β-catenin correlated with increased intercellular adhesion of PANC-1 cells.

**Conclusion:**

These results indicate that active Rac1 destabilises E-cadherin-mediated cell-cell adhesion in pancreatic carcinoma cells by interacting with IQGAP1 which is associated with a disassembly of E-cadherin-mediated adherens junctions. Inhibition of Rac1 activity induced increased E-cadherin-mediated cellular adhesion.

## Background

Strong cell-cell adhesion is a characteristic element of epithelial cells and its assembly and maintenance is part of the cellular differentiation and polarisation process of epithelial cells and tissues. Loss of cellular adhesion accompanied by loss of the underlying protein modules is frequently observed during metastasis of tumours of different origin and results in cellular dedifferentiation and gain of migratory properties of tumour cells [1,2]. The adherens junctions are the best known cell-cell adhesion modules which are responsible for the strong mechanical intercellular adhesion, especially in epithelial tissues. Adherens junctions are mainly built by homophilic interactions of proteins of the cadherin family which are transmembrane calcium-dependent adhesion proteins with E-cadherin as its best characterised member [3].

The association of E-cadherin with the actin cytoskeleton is necessary for strong mechanical cell-cell interaction. This interaction is mediated via E-cadherin-bound β-catenin and α-catenin, which in turn is associated with actin filaments by actin-binding proteins like Eplin, vinculin, formin-1 or α-actinin [4,5]. The assembly of the E-cadherin/catenin adhesion complex is under tight control which involves different posttranscriptional processes like phosphorylation and dephosphorylation, protein interactions and the alteration of protein stability [6]. Moreover, the association of the E-cadherin complex with the actin cytoskeleton is likewise tightly controlled and requires the regulated assembly of actin filaments at sites of cell-cell contacts [7]. The important role of the Rho family member Rac1 in this process has been demonstrated in several reports and is reviewed in [8]. The active, GTP-bound form of Rac1 regulates the formation of submembraneous actin cytoskeleton structures, resulting in the formation of lamellipodia [9]. The localisation of activated Rac1 at membrane sites is a necessary step in the maturation of adherens junctions of epithelial cells during cell polarisation [10]. The molecular mechanism by which Rac1 mediates actin cytoskeletal organisation in epithelial cells is not totally clear yet. The involvement of the Rac1 activator Tiam1, a guanine nucleotide exchange factor, as well as the Rac1 effector IQGAP1 has been documented [11,12]. The IQGAP family comprises a small group of eukaryotic proteins, namely IQGAP1, IQGAP2 and IQGAP3, with IGGAP1 and 2 sharing 62% similarity in the amino acid sequence [13]. IQGAP1 colocalises with actin in lamellipodia and membrane ruffles [14]. Because IQGAP1 and 2 contain several protein interacting domains they are suggested to function as scaffolds coordinating different signalling processes [15]. Several studies have shown the importance of IQGAP proteins for the formation and maturation of E-cadherin-mediated cell-cell adhesion complexes. [16-18]. Moreover, published data confirm an association of IQGAPs with the E-cadherin/catenin adhesion complex [19].

With regard to human cancers pancreatic ductal adenocarcinoma belong to the most fatal cancers because of their very early onset and high frequency of metastasis formation [20]. The molecular mechanism responsible for this extraordinary high rate of metastasis is not known so far. It is documented that Rho GTPases participate in the induction of transformation and metastasis by regulating the organisation of the cytoskeleton as well as gene transcription. [21-23].

In the present manuscript the role of the GTPase Rac1 on E-cadherin-mediated adherens junctions and on cell migration and adhesion was analysed in Rac1(V12)- and Rac1(N17)-expressing PANC-1 cells. We show that enhanced expression of active Rac1(V12) reduces cell-cell adhesion and increases directed cell motility and migration through extracellular matrix, while dominant negative Rac1(N17) mediates the opposite effects. Moreover, activated Rac1(V12) binds to IQGAP1 which is linked to a reduced association of IQGAP1 to β-catenin. These alterations are associated with a disassembly of the E-cadherin/catenin adhesion complex resulting in inhibited cellular aggregation and elevated migratory capacity of pancreatic carcinoma cells. These results suggest that active Rac1 destabilises E-cadherin-mediated cell-cell adhesion in pancreatic carcinoma cells by interacting with IQGAP1 and induction of the disassembly of the E-cadherin/catenin complex. Thus, the data support a role of Rac1 in inhibition of cellular adhesion allowing metastasis formation of pancreatic adenocarcinoma.

## Materials and methods

### Cell culture and transfection

PANC-1 pancreatic carcinoma cells were obtained from ATCC and maintained in DMEM with 10% fetal calf serum (Invitrogen). PANC-1 cells stably transfected with pEGFP/HA-Rac1(V12), pEGFP/Rac1(N17) or pEGFP-C3 (Clontech) were established as described before [24,25]. EGFP- and EGFP-Rac1-expressing cell clones were maintained in growth medium supplemented with 1.5 mg/ml G418 (PAA Laboratories). IQGAP1 protein expression was inhibited by specific double-stranded siRNAs (Qiagen), designed by using Qiagen online siRNA design tool according to the published sequence. Two unrelated siRNA oligonucleotides were used: IQGAP1 si1: gacuuacugcagaggagau; IQGAP1 si2: ugccauggaugagauugga. As control siRNA an oligonucleotide was used without any homology to any known mammalian gene (Qiagen, AllStars neg. Control). Cells were transfected with 66 nM siRNA using DMRIE-C transfection reagent (Invitrogen) and protein expression was analysed after 36-48 h by Western blotting. Cellular proteolytic enzymes were inhibited by treating cultured cells with the permeable pharmacological inhibitors E64 (Roche Diagnostics, 1.5 μM) and MG132 (Calbiochem, 1 μM) for 36 h.

### Antibodies

Monoclonal antibodies against E-cadherin, α- and β-catenin, and Rac1 were obtained from BD Bioscience, monoclonal antibody against GFP was from Roche Diagnostics, polyclonal antibody against β-catenin and monoclonal antibody against β-actin were from Sigma-Aldrich.

### Protein analysis and GST-pull down assays

Cell lysis, SDS gel electrophoresis and Western blotting procedure were performed as described before [23]. Briefly, cells were lysed in NOP buffer [50 mM Tris/HCl (pH 7.2), 1% Triton X-100, 1% NP-40, 150 mM NaCl, containing 400 μM aprotinin, 50 μM leupeptin, and 0.5 mM Pefabloc (all from Roche Diagnostics)] to inhibit proteases. Thirty microgram of total lysates were analysed by SDS-PAGE, blotted onto nitrocellulose and incubated with the indicated antibody over night and a HRP-labelled secondary antibody. Immunoreactive proteins were visualised using an enhanced chemiluminescence detection system (Pierce). Detection of β-actin served to control for equal loading.

For coimmunoprecipitation experiments, 0.5-3 mg of NOP-lysate was used for the μMACS protein isolation system (Miltenyi Biotec) according to manufacture's instructions using the appropriate antibodies. Immunoprecipitates were analysed by Western blotting.

For GST-pull down assays the cDNA of β-catenin was ligated in frame into the GST-vector pGEX-4T1 (GE Healthcare-Amersham), expressed in *E. coli *DH5α and isolated as described before in [26]. Confluent cells were washed with ice-cold TBS (50 mM Tris/HCl, pH 7.5, 150 mM NaCl) and lysed in RIPA buffer (50 mM Tris-HCl pH 7.4, 150 mM NaCl, 1% Triton X-100, 0.5% sodium deoxycholate, 0.5% SDS) including proteinase inhibitors as indicated above. For the assay 500 μg of total protein was mixed with 20-40 μg GST-fusion protein immobilised to glutathione sepharose for 60 min at 4°C. One tenth of the lysate used for precipitation was removed before adding GST-fusion protein and analysed by Western blotting for equal amounts of IQGAP1 and the stably expressed EGFP or EGFP-Rac1 proteins. The amount of IQGAP1 associated with GST-β-catenin was analysed by Western blotting. Representative blots out of three or more independent experiments are shown.

### Immunocytochemical analysis

Cells were seeded on coverslips, fixed in 4% paraformaldehyde and 20% acetic acid for 15 min and permeabilised with 0.1% Triton X-100 in PBS for 10 min for staining of IQGAP1, Rac1 and filamentous actin (F-actin). For the staining of E-cadherin or β-catenin, cells were fixed with ice-cold methanol/acetone 1:1 for 15 min. The first antibodies were applied for 60 min at 37°C followed by a Cy3-coupled secondary antibody (Dianova) for 30 min at 37°C. F-actin was visualised using CPTIC (coumarin phenyl isothiocyanate)-conjugated phalloidin (Sigma-Aldrich). Staining was examined using an IX70 fluorescence microscope (Olympus). Images were processed with AnalySIS 3.2 software (Soft-Imaging System).

### RT-PCR studies

Two-step RT-PCR was performed as described before [27]. RNA was extracted using the RNeasy Mini Kit (Qiagen). To produce cDNA of the used cell clones, 1 μg of total mRNA was transcribed using random primers and Superscript II reverse transcriptase (Invitrogen) in a volume of 50 μl. The semi-quantitative PCR was performed with specific primers for E-cadherin and β-actin using 1 μl of 1/10 diluted cDNA [27].

### Migration, invasion and aggregation studies

Migration, invasion and aggregation assays were performed as described in Vogelmann et al. [28]. The number of cells which had migrated through uncoated (8.0 μm pore size, BD Bioscience) or collagen type I-coated transwell inserts (8.0 μm pore size coated with 70 μl collagen type I solution (0.2 mg/ml, BD Bioscience) was estimated 36 h after seeding of PANC-1 cells stably expressing EGFP, EGFP-Rac1(V12) or EGFP-Rac1(N17). The number of cells which had migrated to the bottom compartment containing DMEM + 10% FCS was estimated by counting three independent, randomly chosen visual fields in a phase contrast microscope (magnification 20×). Three independent assays were performed in duplicate.

To determine the cell-cell adhesion capacity, cell rotation aggregation assays were performed as described in [28]. The aggregation index was calculated from the ratio of the aggregate number after 30 min of rotation and the particle number at the beginning (A_i _= (N_0_-N_30_)/N_0_). Five independent assays were performed in duplicate.

### Subcellular fractionation

For subcellular fractionation into soluble (S100) and particulate membrane-containing (P100) fractions, confluent cells were scraped into 500 μl HEPES buffer [50 mM HEPES, pH 7.6, 8.6% (m/v) sucrose, 10 mM EDTA, 10 mM EGTA, 1 mM Pefabloc, 40 μg/μl leupetin, 0.4 mg/ml soybean trypsin inhibitor, 40 μg/ml pepstatin A] and fractionated by high speed centrifugation as described in [23]. To obtain Triton X-100-soluble and -insoluble fractions, cells were incubated with Triton-lysis buffer (1% Triton X-100, 0.3 M sucrose, 25 mM HEPES, pH 7.4, 100 mM NaCl, 4.7 mM KCl, 1.2 mM KH_2_PO_4_, 1.2 mM MgCl_2_, 5 μM aprotinin, 1 mM Pefabloc, 5 μM soybean trypsin inhibitor) for 15 min on a rocking platform as described in [28]. The Triton-soluble and the Triton-insoluble fractions were reconstituted to equal volumes and 15 μl of each fraction were analysed by Western blotting.

### Rac1 activity assay

The amount of GTP-bound Rac1 was determined by using the CRIB-domain of PAK1B (GST-PAK) as an activation-specific probe for activated Rac1 as described in [25].

### Statistics

To analyse statistical differences means and standard error means (SEM) were compared using the unpaired student t-test and Prism 4 software (GraphPad Software, Inc.).

## Results

### Expression of EGFP-Rac1(V12) and EGFP-Rac1(N17) in pancreatic carcinoma cells

The aim of this study was to characterise the function of Rac1 in epithelial cell differentiation and migration, especially during carcinogenesis of pancreatic cancer cells. Therefore, we stably expressed constitutively active EGFP (enhanced green fluorescent protein)-Rac1(V12) or dominant negative EGFP-Rac1(N17) in the pancreatic carcinoma cell line PANC-1. PANC-1 clones stably expressing EGFP served as control. The expression of EGFP and EGFP-Rac1 proteins was analysed by immunoblotting of lysates of the obtained cell clones and 2-4 clones for each protein were selected for further investigation. Approximately 90% of cells of the selected clones showed EGFP or EGFP-Rac1 expression. Since the number of EGFP-Rac1-expressing cells decreases in the course of long term culture, the number of fluorescent cells was controlled regularly. If necessary the culture was replaced by a culture of a low passage number to keep high numbers of EGFP-Rac1-expressing cells. Data of one representative cell clone expressing EGFP [clone EGFP-21], EGFP-Rac1(V12) [clone Rac1(V12)-10.1] or EGFP-Rac1(N17) [clone Rac1(N17)-2] are shown in all figures.

**Figure 1:**
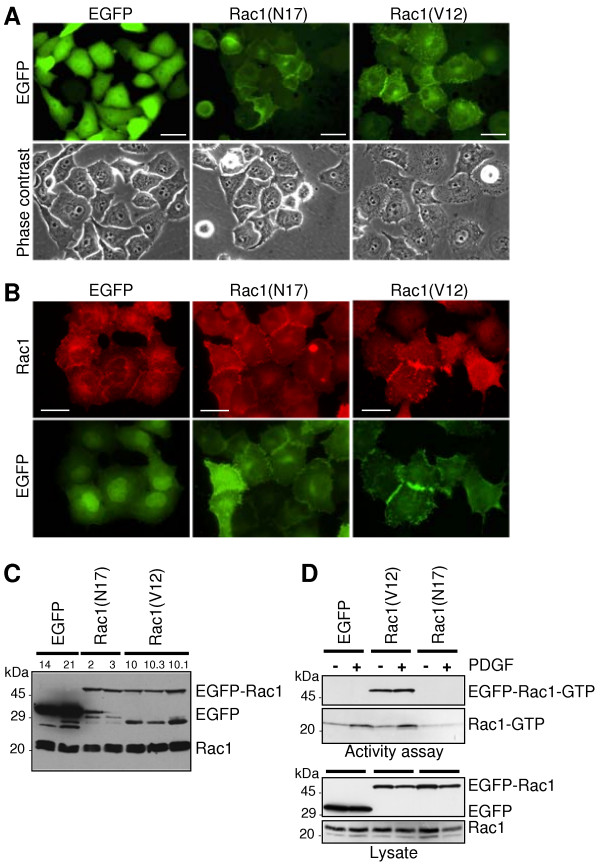
**Expression of EGFP-Rac1(V12) and EGFP-Rac1(N17) in PANC-1 cells. (A) **Localisation of EGFP-Rac1(V12) and EGFP-Rac1(N17). EGFP fluorescence (upper panel) and phase contrast analyses (lower panel) demonstrate the localisation of EGFP proteins and the morphology of subconfluent, living PANC-1 cells stably expressing EGFP, EGFP-Rac1(V12) and EGFP-Rac1(N17), bar = 20 μm. **(B) **Immunofluorescence analysis of Rac1 localisation. Fixed EGFP-, EGFP-Rac1(V12)- and EGFP-Rac1(N17)-expressing cells were incubated with Rac1-specific antibody. EGFP fluorescence is illustrated for comparison (bar = 30 μm). **(C) **Expression of EGFP, EGFP-Rac1 and endogenous Rac1 in 30 μg of total cell lysate of different cell clones was estimated by Western blotting using Rac1- and GFP-specific antibodies. One representative blot of three independent experiments is shown. **(D) **The amount of active Rac1-GTP in EGFP- and EGFP-Rac1-expressing cells was determined by affinity precipitation assays (upper panel). The cells were serum-starved for 24 h and then left untreated (-) or treated with 10 ng/ml PDGF AB for 5 min (+). The assay was performed with 500 μg (Rac1) or 1000 μg (EGFP-Rac1) of cell lysate. To control for equal loading, aliquots of the samples were analysed in parallel in Western blotting (lower panel). One representative blot out of three independent experiments is shown.

Figure 1A shows the localisation of the ectopically expressed EGFP and EGFP-Rac1 proteins and documents that both Rac1 mutants were equally expressed. The dominant negative EGFP-Rac1(N17) is mainly localised at the cell membrane and is concentrated in areas of cell-cell contacts (Figure 1A). The membranous localisation of EGFP-Rac1(N17) was confirmed by subcellular fractionation of total cell lysate in a soluble fraction containing cytosolic proteins and a particulate, membrane-containing fraction (as demonstrated later in Figure six A). The stably expressed constitutively active EGFP-Rac1(V12) was localised in the membrane and the cytoplasm of the cells (Figure 1A). Immunofluorescence staining of endogenous Rac1 (Figure 1B) demonstrate that endogenous Rac1 is localised at the plasma membrane and in the cytoplasm of PANC-1 cells. This distribution was also evident after subcellular fractionation and Western blotting shown later in Figure six A. The obtained results are in agreement with the localisation of Rac1 and EGFP-Rac1 mutants in MDCK and COS1 cells [29].

Western blot analyses confirmed the expression of Rac1 fusion proteins of the expected size (approximately 50 kDa for EGFP-Rac1 and 30 kDa for EGFP) (Figure 1C) as exemplified for two EGFP-Rac1(N17) and three EGFP-Rac1(V12) cell clones. Two EGFP-expressing clones served as controls. The various bands seen at a molecular weight < 30 kDa are most likely degradation products of EGFP-Rac1 detected by the GFP antibody. The amount of endogenous Rac1 was not altered by the expression of the EGFP-Rac1 mutants. To analyse the activity of endogenously and ectopically expressed Rac1 in serum-starved and PDGF AB-treated cells, affinity precipitation assays were performed using the Rac1-binding domain of PAK1B as an activation-specific probe to isolate Rac1-GTP. As demonstrated in Figure 1D only EGFP-Rac1(V12) was precipitated and therefore active, whereas dominant negative EGFP-Rac1(N17) was not precipitated (Figure 1D). Treatment of the cells with 10 ng/ml PDGF AB for 5 min resulted in activation of endogenous Rac1 in the EGFP-expressing control cells and the EGFP-Rac1(V12)-expressing cells, whereas the constitutively active EGFP-Rac1(V12) was not further activated. The activity of endogenous Rac1 in cells expressing the dominant negative EGFP-Rac1(N17), which is reduced as compared to the activity in EGFP- and EGFP-Rac1(V12)-expressing cells, was not increased by PDGF AB, demonstrating the inhibitory effect of this mutant on Rac1 activation.

### Rac1(V12) decreases E-cadherin adhesion complexes in PANC-1 cells

**Figure 2:**
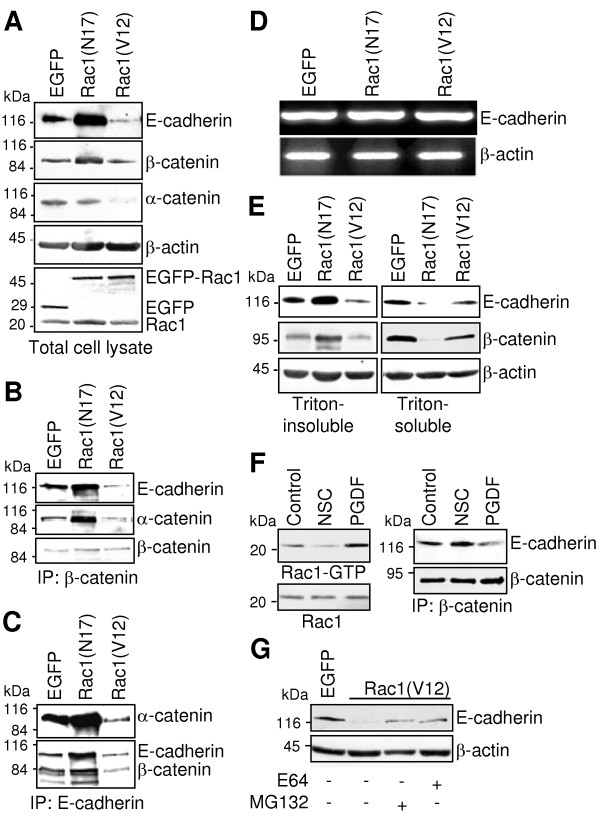
**EGFP-Rac1-induced differences in the amount of cell-cell adhesion protein concentration**. **(A) **The amount of E-cadherin, α- and β-catenin in 30 μg of total cell lysate was analysed by Western blotting. Individual proteins were detected by using specific antibodies. Detection of β-actin served as control to document equal amounts of protein in each lane.**(B+C) **The amount of the E-cadherin/catenin complex was analysed by immunoprecipitation of β-catenin **(B) **or E-cadherin **(C) **from 300 μg of total cell lysate. Coprecipitated E-cadherin and α-catenin **(B) **or α-catenin and β-catenin **(C)**, respectively, were identified by Western blotting. **(D) **Reverse transcription-PCR analysis was performed using total RNA isolated from PANC-1 cells stably expressing EGFP, EGFP-Rac1(N17) or EGFP-Rac1(V12). E-cadherin mRNA was amplified using specific primers. Amplification of β-actin cDNA served as control to document equal amounts of mRNA. **(E) **Association of the E-cadherin/catenin complex with the actin cytoskeleton was analysed using Triton X-100-fractionated cell extracts. Amounts of E-cadherin and β-catenin were analysed by Western blotting using 10 μg of Triton X-100-soluble and Triton X-100-insoluble fraction. **(F) **To investigate the influence of endogenous active Rac1 on the E-cadherin/catenin complex, PANC-1 cells were treated with 50 μM of the Rac1-inhibitor NSC23766 or stimulated for 5 min with PDGF AB (10 ng/ml). Active Rac1-GTP was precipitated from 800 μg of cell lysates by affinity precipitation assays (upper panel). To control for equal loading, aliquots of the samples were analysed in parallel (lower panel). The amount of E-cadherin/catenin complexes was analysed by detection of E-cadherin, which was coprecipitated with β-catenin from 500 μg of total cell lysates. **(G) **To investigate E-cadherin degradation in Rac1(V12)-expressing PANC-1 cells, cells were incubated with MG132 (1 μM) or E64 (1.5 μM) to suppress proteolytic degradation of proteins. The amount of E-cadherin was estimated by Western blotting using 30 μg of total protein lysates. Determination of β-actin served to control for comparable loading. In each figure one representative blot/agarose gel of three to four independent experiments is shown.

Because EGFP-Rac1 was localised in areas of cell-cell contacts which might interfere with the E-cadherin-mediated cell-cell adhesion, the effects of Rac1 expression on the intercellular adhesion was analysed. First, the concentration of E-cadherin/catenin complex proteins was comparatively analysed in total cell lysates of EGFP-, EGFP-Rac1(N17)- and EGFP-Rac1(V12)-expressing PANC-1 cells. As shown in Figure 2A, PANC-1 cells expressing EGFP-Rac1(N17) exhibited increased concentrations of E-cadherin and β-catenin and equal amounts of α-catenin in total cell lysates compared to EGFP-expressing controls. In contrast, cells expressing constitutively active EGFP-Rac1(V12) showed dramatically reduced amounts of E-cadherin and α-catenin and slightly reduced amounts of β-catenin (Figure 2A). Detection of EGFP and Rac1 confirmed comparable amounts as well as the presence and correct size of ectopically expressed EGFP and EGFP-Rac1 as well as endogenous Rac1 (Figure 2A). Staining of β-actin served to control for equal amounts of protein used in each lane. To determine whether the increased amount of E-cadherin was due to enhanced gene expression, the amount of E-cadherin mRNA was analysed by RT-PCR. As demonstrated in Figure 2D only minor differences in the amount of the E-cadherin mRNA were detected between the analysed cell clones pointing to a regulation of the amount of E-cadherin on the protein level.

To achieve intercellular adhesion, E-cadherin needs to be assembled in cadherin/catenin complexes. To estimate the amount of E-cadherin/catenin complexes present in EGFP-Rac1-expressing PANC-1 cells, coimmunoprecipitation experiments were performed. Immunoprecipitation of equal amounts of β-catenin and staining of coprecipitated E-cadherin and α-catenin revealed a significant increase in the amount of coprecipitated E-cadherin and α-catenin in cells expressing EGFP-Rac1(N17) compared to EGFP-expressing controls (Figure 2B). In contrast, reduced amounts of E-cadherin and α-catenin were associated with β-catenin in EGFP-Rac1(V12)-expressing PANC-1 cells, indicating reduced amounts of E-cadherin adhesion complexes (Figure 2B). These changes were confirmed by immunoprecipitating E-cadherin and detection of associated α- and β-catenin (Figure 2C).

Furthermore, the association of the cadherin/catenin complex with the actin cytoskeleton is indispensable for strong cell-cell interactions. To analyse the amount of E-cadherin/catenin complexes which are associated with the cytoskeleton, the distribution of E-cadherin, α- and β-catenin was determined in Triton X-100-fractionated cell lysates. Triton X-100 fractionation separates cytoskeletal and cytoskeleton-associated proteins, which are present in the Triton-insoluble fraction, from soluble and cytoplasmic proteins, enclosed in the Triton-soluble fraction [30]. As demonstrated in Figure 2E, expression of the constitutively active EGFP-Rac1(V12) correlated with reduced levels of cytoskeleton-bound E-cadherin, whereas the expression of dominant negative EGFP-Rac1(N17) resulted in an increased amount of Triton-insoluble, cytoskeleton-bound E-cadherin and β-catenin (Figure 2E). The decrease in the amount of E-cadherin and β-catenin in the Triton-soluble fraction of the EGFP-Rac1(N17)-expressing cells suggests a stabilising effect of the dominant negative Rac1 mutant on the E-cadherin complex and its association with the cytoskeleton.

Next, we analysed whether inhibition or activation of endogenous Rac1 provokes similar effects in the E-cadherin adhesion complex. Therefore, Rac1 activity was either inhibited by treatment of PANC-1 cells with a Rac1 inhibitor NSC23766 (50 μM) [31] for 16 h or stimulated by incubation of the cells with the growth factor PDGF AB [32] for 5 min. Rac1 activity assays (Figure 2F) demonstrate that the Rac1 inhibitor suppressed the activity of endogenous Rac1 in PANC-1 cells. PDGF AB enhanced the amount of active Rac1, thereby verifying the appropriate effects of the two substances. Next, the amount of E-cadherin which coprecipitated with β-catenin was analysed by coimmunoprecipitation and Western blot procedure. As shown in Figure 2F, activation of Rac1 by PDGF-treatment clearly reduced the concentration of the E-cadherin/catenin complexes, as demonstrated by the reduced E-cadherin amount. However, inhibition of Rac1 by treatment with the Rac1 inhibitor for 16 h increased the amount of E-cadherin which was coprecipitated with β-catenin. These data are in agreement with the results shown before, which demonstrated a detachment of E-cadherin from the actin cytoskeleton in PANC-1 cells after ectopic expression of constitutively active Rac1(V12) and an increased association after expression of dominant negative Rac1(N17). The lower amount of E-cadherin in EGFP-Rac1(V12)-expressing cells was not due to reduced gene expression as shown above. Thus, we asked whether an increased degradation of E-cadherin may be responsible for the altered E-cadherin level. EGFP-Rac1(V12)-expressing PANC-1 cells were treated with the proteolytic inhibitors E64 and MG132 for 36 h. The Western blots demonstrated in Figure 2G, clearly show increased amounts of E-cadherin in inhibitor-treated EGFP-Rac1(V12) cells. Therefore, these results points to an elevated proteosomal degradation of E-cadherin in EGFP-Rac1(V12)-expressing PANC-1 cells.

### Activated Rac1 inhibits cell aggregation and promotes cell migration

**Figure 3:**
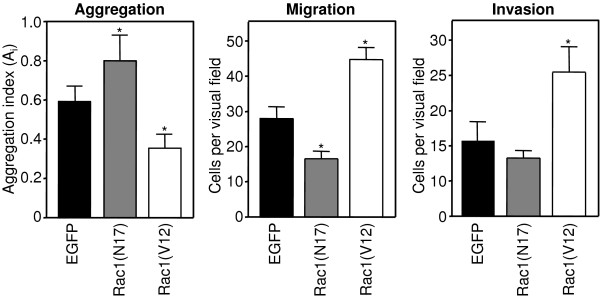
**Rac1 influences the cell aggregation and cell migration of PANC-1 cells**. Cell-cell adhesion of PANC-1 cells stably expressing EGFP, EGFP-Rac1(V12) or EGFP-Rac1(N17) was analysed by rotation aggregation assays. Cell migration was studied using uncoated transwell migration assays. The number of migrated cells in three randomly chosen visual fields (magnification 20×) is given. Cell migration through extracellular matrix was analysed using collagen type I-coated transwell inserts. Mean +/- SEM of three independent assays performed in duplicate is shown. Statistical differences were analysed compared to EGFP-expressing controls using the unpaired student t-test and (*) p < 0.05 was considered significant.

Next, we investigated the consequence of the Rac1-modulated E-cadherin/catenin amounts and complex stability on cell-cell adhesion, cell migration and invasion of PANC-1 cells stably expressing EGFP, EGFP-Rac1(V12) or EGFP-Rac1(N17). The influence of Rac1 on cell-cell adhesion was determined by rotation aggregation assays. As shown in Figure 3, dominant negative Rac1(N17) enhanced the cellular aggregation of PANC-1 cells statistically significant (p < 0.02, Student t-Test) compared to the control cells. In contrast, constitutively active Rac1(V12) significantly reduced the cell aggregation capacity of PANC-1 cells (p < 0.05, Student t-Test). These data demonstrate that the Rac1-induced alteration in the amount and association of the E-cadherin/catenin complex reported above are reflected by changes in cell-cell adhesion.

Because a reduction of cell-cell adhesion is frequently observed in migrating epithelial cells, the influence of the Rac1 mutants was analysed on cellular motility. Directed cell migration was studied using uncoated transwell cell migration chambers. Figure 3 demonstrates that PANC-1 cells expressing dominant negative EGFP-Rac1(N17) and therefore increased amounts of E-cadherin adhesion complexes, exhibit a significant reduction of cell migration as compared to the EGFP-expressing control cells. Expression of EGFP-Rac1(V12) resulted in a nearly two-fold increase in the number of migrated cells compared to EGFP-transfected PANC-1 cells (Figure 3). The same tendency was observed when collagen type I-coated chambers were used. EGFP-Rac1(V12)-expressing cells exhibited a markedly increased cell invasion through collagen I matrix, whereas EGFP-Rac1(N17)-expressing cells showed a slightly inhibited cell invasion through the collagen matrix (Figure 3).

### Rac1(V12) alters the localisation of IQGAP1

**Figure 4:**
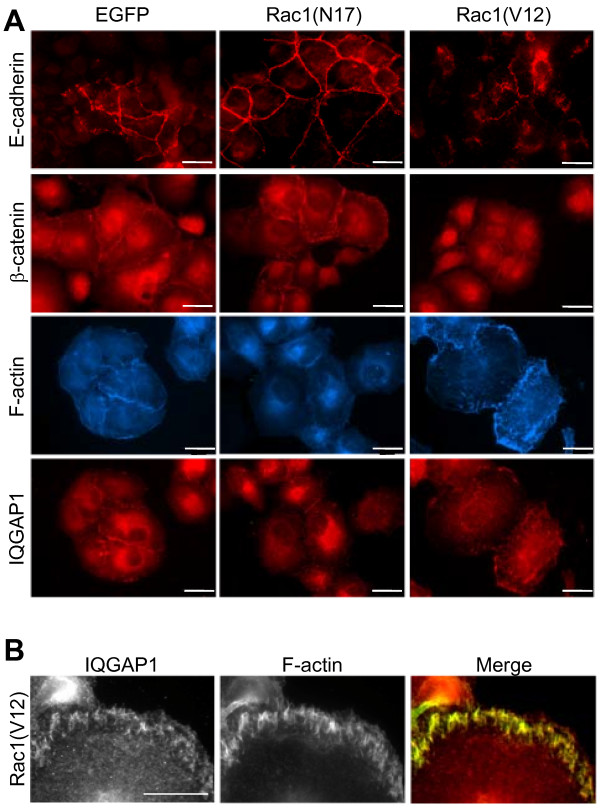
**Immunofluorescence localisation of E-cadherin, β-catenin, IQGAP1 and filamentous actin (F-actin)**. **(A) **PANC-1 cells stably expressing EGFP, EGFP-Rac1(V12) or EGFP-Rac1(N17) were incubated with specific antibodies against E-cadherin, β-catenin, IQGAP1 or CPTIC-conjugated phalloidin. The staining was examined by fluorescence microscopy (bar = 20 μm). **(B) **EGFP-Rac1(V12)-expressing PANC-1 cells were stained for IQGAP1 and F-actin with CPTIC-conjugated phalloidin. The merged image shows in yellow the colocalisation of IQGAP1 and filamentous actin (bar = 10 μm).

The changes in the amount of E-cadherin/catenin complexes and the subsequently increased cell migration correlates with the localisation of E-cadherin, β-catenin and filamentous actin, as determined by immunofluorescence analyses (Figure 4A). EGFP-Rac1(N17)-positive PANC-1 cells showed enhanced E-cadherin staining as well as enhanced β-catenin staining, which predominantly localised in areas of cell-cell-contacts. The Rac1(V12)-expressing cells showed a reduced and punctuated E-cadherin staining and diminished β-catenin fluorescence compared to EGFP- and EGFP-Rac1(N17)-expressing cells, especially in the areas of cell-cell contacts. This redistribution of β-catenin correlated with changes in the organisation of actin filaments. A loss of cortical actin and an increase in short actin filaments beneath the plasma membrane was observed in EGFP-Rac1(V12)-expressing PANC-1 cells (Figure 4A and 4B).

Most interestingly, the Rac1 effector IQGAP1, formerly described as a GTPase activating protein (GAP) for Rac [33], also showed a differential localisation in PANC-1 cells depending on Rac1 activity. In the EGFP-expressing controls as well as in EGFP-Rac1(N17)-expressing cells, IQGAP1 was localised mainly at or near the plasma membrane and was concentrated in areas of cell-cell contacts (Figure 4A). In PANC-1 cells expressing constitutively active EGFP-Rac1(V12) IQGAP1 colocalised with the short actin filaments (Figure 4A and 4B). This colocalisation of IQGAP1 and F-actin becomes evident in the enlarged micrographs and the merged image shown in Figure 4B, in which the yellow colour indicates similar localisation of the two proteins.

### Rac1(N17) promotes the interaction between β-catenin and IQGAP1 in PANC1-cells

**Figure 5:**
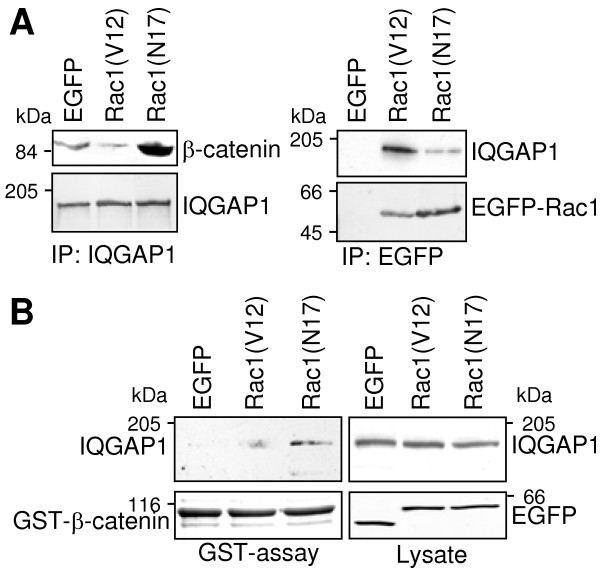
**Interaction of IQGAP1 with Rac1 or β-catenin. (A) IQGAP1 was precipitated from lysates of EGFP-, EGFP-Rac1(V12)- and EGFP-Rac1(N17)-expressing PANC-1 cells and coprecipitated β-catenin was determined by Western blotting (left panel)**. In parallel, immunoprecipitates of EGFP/EGFP-Rac1 proteins were analysed for coprecipitated IQGAP1 (right panel). Staining of the precipitated proteins verified equal amounts of protein in each immunoprecipitation (lower panels) **(B) **Binding of IQGAP1 to β-catenin was confirmed by GST-pull down assays. GST-tagged β-catenin was incubated with 500 μg of lysate prepared from EGFP-, EGFP-Rac1(V12)- or EGFP-Rac1(N17)-expressing PANC-1 cells and GST-β-catenin complexes were precipitated by binding to glutathione sepharose. Precipitated IQGAP1 and GST-β-catenin was determined by Western blotting. Determination of IQGAP1 and EGFP proteins in 30 μg of the cell lysate served to demonstrate equal amounts of protein in each sample. One representative blot of three independent experiments is shown.

The different localisation of IQGAP1 observed in immunofluorescence studies of PANC-1 cells harbouring different Rac1 mutants was further analysed in coimmunoprecipitation experiments. IQGAP1 was coimmunoprecipitated with β-catenin from lysates of cells expressing dominant negative EGFP-Rac1(N17) (Figure 5A). In cells expressing constitutively active EGFP-Rac1(V12) only small amounts of β-catenin were coprecipitated with IQGAP1 (Figure 5A, left panel). But immunoprecipitation of EGFP-Rac1 resulted in the case of EGFP-Rac1(V12) in high amounts of coprecipitated IQGAP1 (Figure 5A, right panel) in contrast to precipitation of EGFP-Rac1(N17) where only small amounts of IQGAP1 were coprecipitated (Figure 5A, right panel).

The interaction between β-catenin and IQGAP1 was confirmed by *in vitro *binding experiments. Bacterially expressed GST-β-catenin was incubated with cell lysates of PANC-1 cells stably expressing EGFP, EGFP-Rac1(V12) or EGFP-Rac1(N17). IQGAP1 was purified by GST-β-catenin only from lysates of cells expressing EGFP-Rac1(N17) but not form lysates of EGFP-Rac1(V12)-expressing cells (Figure 5B). Aliquots of the used cell lysates were analysed to confirm the presence of equal amounts of IQGAP1 and of the EGFP-proteins (Figure 5B).

### IQGAP1 interacts with β-catenin in the membrane fraction of Rac1(N17)-expressing pancreatic epithelial cells

**Figure 6:**
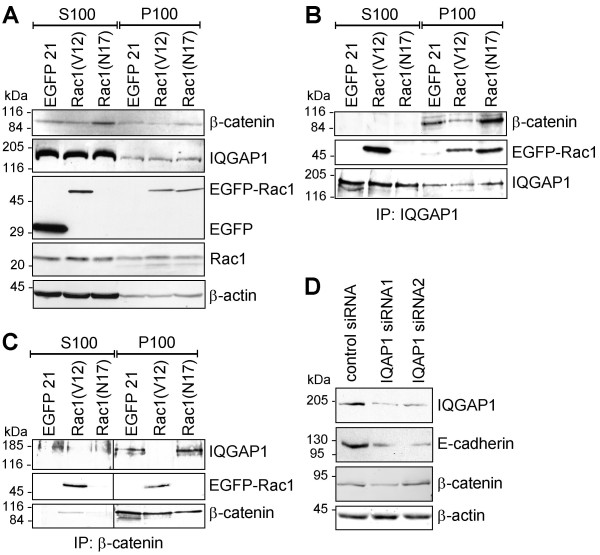
**Analyses of Rac1/IQGAP1/β-catenin complexes in EGFP-Rac1(V12)- or EGFP-Rac1(N17)-expressing PANC-1 cells**. **(A) **Soluble (S100) and particulate (P100) fractions of EGFP-, EGFP-Rac1(V12)- and EGFP-Rac1(N17)-expressing PANC-1 cells were prepared from total cell lysates by centrifugation at 100000 × g. Subcellular localisation of Rac1, IQGAP1 and β-catenin was analysed in aliquots of 50 μg by Western blotting. To control for comparable loading β-actin was determined (lower panel). **(B) **Immunoprecipitation was performed using 300 μg of soluble (S100) or particulate, membrane-containing (P100) fractions of PANC-1 cells stably expressing EGFP, EGFP-Rac1(V12) or EGFP-Rac1(N17). After immunoprecipitation of IQGAP1 coprecipitated β-catenin and EGFP-Rac1 was determined by Western blotting. Detection of the immunoprecipitated IQGAP1 demonstrated equal amounts of protein in each precipitation. **(C) **Immunoprecipitation of β-catenin was performed in parallel experiments. Coprecipitated IQGAP1 and EGFP-Rac1 was determined by Western blotting and detection of the immunoprecipitated β-catenin verified equal amounts of protein. **(D) **The concentration of IQGAP1 in PANC-1 cells was reduced by transfection of two different siRNA oligonucleotides targeting IQGAP1. The amount of E-cadherin, β-catenin and IQGAP1 was demonstrated by Western blotting. Staining of β-actin served to demonstrate equal amounts of cell lysate. In each figure one representative blot out of three independent experiments is shown.

In order to determine the localisation and composition of the IQGAP1-, β-catenin-, Rac1-complexes in more detail coimmunoprecipitation studies with subcellular fractions of cell lysates were performed. Soluble (S100) and particulate (P100) fractions of EGFP-, EGFP-Rac1(V12)- and EGFP-Rac1(N17)-expressing PANC-1 cells were obtained by high speed centrifugation at 100000 × g. The particulate fraction contains membranes and membrane-associated proteins and the soluble fraction enclose cytoplasmic proteins. Figure 6A shows the distribution of IQGAP1, β-catenin and Rac1 proteins in the two subcellular fractions of the different PANC-1 cell clones. IQGAP1 and endogenous Rac1 were present in both the S100 and the P100 fraction and this distribution was not influenced by the expression of EGFP-Rac1(V12) or EGFP-Rac1(N17). β-catenin was also present in both S100 and P100 fractions, but higher amounts were detectable in the fractions prepared from EGFP-Rac1(N17)-expressing PANC-1 cells as compared to the controls. This increase is in agreement with the enhanced amount of β-catenin in total lysates of EGFP-Rac1(N17)-expressing cells. The EGFP-Rac1 mutants showed a differential distribution. Whilst EGFP-Rac1(N17) was detected only in the particulate, membrane containing P100 fraction, EGFP-Rac1(V12) was localised in both the S100 and the P100 fraction. These results indicate that the distribution of the constitutive active and the dominant negative mutant of Rac1 is differentially regulated in the cells. The coimmunoprecipitation experiments, shown in Figure 6B and 6C, led to the following results. IQGAP1-immunoprecipitates revealed that β-catenin was only coprecipitated from the particulate, membrane-containing fraction. Interestingly, the highest amount of IQGAP1/β-catenin complexes was precipitated from the particulate fraction of EGFP-Rac1(N17)-expressing cells, whereas only minor quantity of β-catenin was coprecipitated with IQGAP1 from the particulate fraction of EGFP-Rac1(V12)-expressing cells (Figure 6B upper panel). Moreover, when the amount of EGFP-Rac1 which coprecipitated with IQGAP1 (Figure 6B middle panel) was analysed, it was obvious that only membrane-associated EGFP-Rac1(N17) interacted with IQGAP1. In contrast, EGFP-Rac1(V12)/IQGAP1 complexes were determined in both the particulate and to a higher amount in the soluble fraction (Figure 6B). These results demonstrate that IQGAP1 interacts on the one hand with β-catenin and EGFP-Rac1(N17) in the membrane-containing fraction and on the other hand markedly with constitutively active EGFP-Rac1(V12) in the soluble fraction (Figure 6B). To verify these coimmunoprecipitation data, β-catenin was precipitated from the S100 and the P100 fractions and the precipitates were analysed for coprecipitated IQGAP1 and EGFP-Rac1 by Western blotting. The obtained results are shown in Figure 6C. In the particulate, membrane-containing fraction, β-catenin was associated with IQGAP1 in lysates from EGFP- and EGFP-Rac1(N17)-expressing cells but not from EGFP-Rac1(V12)-expressing cells. An interaction of β-catenin with EGFP-Rac1(V12) was found in the soluble as well as in the particulate fraction of EGFP-Rac1(V12)-expressing cells (Figure 6C). In the experiments exemplified in Figure 6C a protein complex containing all three proteins, namely IQGAP1, Rac1 and β-catenin was not detectable. In summary, these data suggest the presence of two different pools of IQGAP1 complexes. One complex contains IQGAP1 and β-catenin and is localised in the membrane fraction of inactive Rac1(N17)-expressing PANC-1 cells, whereas the second is composed of IQGAP1 and active Rac1 and is mainly situated in the soluble fraction of Rac1(V12)-expressing epithelial cells.

The hypothesis that IQGAP1 stabilises the amount of E-cadherin as well as the E-cadherin/catenin complex was supported by experiments in which IQGAP1 expression was downregulated by specific siRNA. The siRNA clearly downregulated the amount of IQGAP1 on protein level after 48 h (Figure 6D). Moreover, the E-cadherin protein level was reduced significantly (Figure 6D), whereas the amount of β-catenin was not altered after the knock-down of IQGAP1, indicating a crosstalk of IQGAP1 and E-cadherin.

## Discussion

In the present study we have analysed the influence of the Rho-GTPase Rac1 on E-cadherin-mediated cell-cell adhesion in the pancreatic carcinoma cell line PANC-1. An enhanced expression of Rac1 has been documented for pancreatic cancer tissue and pancreatic carcinoma cell lines [34] and active Rac1 is necessary for cell migration and invasion in pancreatic tumours after HGF or LPA-stimulation [25,35]. Although many data have been collected with regard to a role of Rac1 proteins in the regulation of epithelial cell polarity as well as of cell-cell adhesion complexes [36], the molecular function of Rac1 in apico-basal determination of cell polarity is still controversial. [37-40]. By using pancreatic carcinoma cell clones stably expressing constitutively active Rac1(V12), which is constantly in its GTP-bound active form, and dominant negative Rac1(N17), which competes with normal Rac1 for binding to guanine nucleotide exchange factors but cannot interact with downstream effector proteins [29,41], we demonstrate in the present manuscript the following findings: First, constitutively active Rac1 downregulates E-cadherin, α-catenin and slightly β-catenin protein content and the amount of E-cadherin/catenin adhesion complexes, inhibits E-cadherin-mediated cell-cell aggregation and supports cellular migration and invasion. Second, expression of dominant negative Rac1(N17) results in increased amounts of E-cadherin as well as β-catenin in pancreatic carcinoma cells, which correlated with a stabilisation of E-cadherin/catenin adhesion complexes resulting in increased cell-cell adhesion. These findings points to the ability of endogenous Rac1 to modulate E-cadherin and its function. The inhibition of endogenous Rac1 activity by a Rac1-specific inhibitor also increased the amount of E-cadherin/β-catenin complexes. Whereas activation of Rac1 by PDGF-treatment reduced the amounts of E-cadherin/β-catenin complexes, thereby supporting that the E-cadherin adhesion complex is controlled by Rac1. Pharmacological inhibition of proteasome-mediated E-cadherin degradation partially reverted the effect of Rac1(V12) on E-cadherin protein content, indicating the E-cadherin protein turnover but not its gene expression is influenced by active Rac1 (Figure 2D+2G).

The destabilising effect of activated Rac1(V12) on cell-cell adhesion is in agreement with studies obtained in human keratinocytes by V. Braga and colleagues demonstrating that microinjection of constitutively active Rac1(V12) induced a dissociation of E-cadherin from the intercellular junctions [37,42]. Experiments by Lozano and coworkers showed that the p21 activated kinase 1 (PAK1), a Rac1 effector, is also involved in the regulation of E-cadherin-mediated cell-cell adhesion. Expression of activated Rac1 or activated PAK1(K299R) in keratinocytes leads to destabilisation of the E-cadherin/catenin complexes [43]. In addition, these data are supported by findings of Pujuguet et al. which illustrate that phosphorylated ezrin or the ezrin mutant T567D, which stimulated Rac1 activation in MDCK cells, induced internalisation and degradation of E-cadherin [44]. In contrast, expression of dominant negative Rac1(N17) stabilised E-cadherin concentration as well as its localisation in areas of cell-cell contacts [44]. It has been shown that clustering of endogenous E-cadherin leads to activation of Rac1 in some cell types which is necessary to form a primordial E-cadherin-mediated adhesion. However, Rac1 activation is not necessary for the maturation of these primordial adhesions to the well described differentiated adherens junction of epithelial cells [39,45], beyond that Rac1 activity tend to inhibit differentiation of adherens junctions [10].

On the molecular level our data suggest that the Rac1-effector IQGAP1 [46] mediates at least some effects described in this study. A role for IQGAP1 in Rac1-mediated regulation of E-cadherin adhesion complexes using E-cadherin-transfected fibroblasts has been demonstrated before by Kuroda and colleagues [47,48]. In the present manuscript immunofluorescence analyses showed enhanced E-cadherin and β-catenin staining in areas of cell-cell contacts of Rac1(N17)-expressing PANC-1 cells and a concentration of IQGAP1 in these areas. Moreover, IQGAP1 strongly interacts with β-catenin in Rac1(N17)-expressing PANC-1 cells. The coimmunoprecipitation of E-cadherin with IQGAP1 (data not shown) argues for an interaction of IQGAP1 with the E-cadherin complex via β-catenin. The stabilising function of IQGAP1 on the turnover of E-cadherin/catenin complexes was underlined by the downregulated IQGAP1 expression by siRNAs which resulted in a disassembly of the E-cadherin/β-catenin complex and reduced concentration of E-cadherin in the analysed pancreatic carcinoma cells. A recent report by Brown and coworkers demonstrated that microinjection of IQGAP1 mRNA into early *Xenopus *embryos resulted in loss of cell-cell adhesion in the late blastula stage. In *Xenopus *the destabilising effect of IQGAP1 depends on its interaction with the GTPase Cdc42 and is independent of Rac1, which represents an example for another aspects of IQGAP1 function [11].

β-catenin did not interact with IQGAP1 in the Rac1(V12)-expressing cells, in which IQGAP1 colocalised with the short actin filaments just under the plasma membrane, where it might interact with F-actin [49]. This reduced interaction between IQGAP1 and β-catenin is associated with destabilised cell-cell adhesion of PANC1 pancreatic carcinoma cells due to reduced amounts of E-cadherin/catenin complexes. With regard to the current knowledge, one can speculate that this reduction is likely a consequence of decreased protein half life time of E-cadherin due to an elevated turnover of E-cadherin/catenin complexes [50]. The elevated turnover was suggested to be caused by a reduced attachment of the E-cadherin complex with the actin cytoskeleton in Rac1(12)-expressing PANC-1 cells [50]. Interestingly, IQGAP1/EGFP-Rac1(V12) complexes were found in the membrane-containing and to a higher content in the cytoplasmic fraction of PANC-1 cells, whereas IQGAP1/EGFP-Rac1(N17) complexes were only detectable in the membrane-containing fraction. EGFP-Rac1(V12) was localised in the membrane-fraction and in the cytosol, which resembles the localisation of endogenous Rac1. Several reports have demonstrated that most of the Rac1 proteins are localised in the cytoplasm of quiescent cells, due to a high-affinity interaction with Rho guanine dissociation inhibitors (RhoGDIs), especially RhoGDIα [51,52]. Activation of Rac1 leads to dissociation of the Rac1/GDI complex and translocation of Rac1 to the membrane [53]. Although EGFP-Rac1(V12) is constantly in its GTP-bound form the V12-mutant of Rac1 can still interact with GDIs, which results in its targeting to the cytoplasmic compartment as well. However, Rac1(N17) mutants, although locked in the inactive conformation, have been demonstrated exclusively in the membrane compartment (this study and [29]). This is explained by its failure to interact with RhoGDIα. It has been shown that dominant negative mutants of Rho GTPases exist in a nucleotide-free state which favours an "unproductive" interaction with GEFs at membrane compartments (discussed in [29]). The coimmunoprecipitation analyses performed in this study revealed two pools of IQGAP1 complexes. IQGAP1 is associated with β-catenin in the membrane of PANC-1 cells with low Rac1 activity where it stabilises E-cadherin/β-catenin complexes. Moreover, IQGAP1 is associated with active Rac1 in the soluble fraction of epithelial PANC-1 cells, which exhibits only small amounts of E-cadherin/β-catenin complexes. Association of IQGAP1 with active Rac1 stabilises its active conformation [54]. Previous discoveries emphasise that Rac1 controls the subdistribution of IQGAP1 in the way that reduction of Rac1-GTP levels decreases Rac1/IQGAP1 levels and favours the formation of IQGAP1/β-catenin complexes which inhibits intercellular adhesion of E-cadherin-transfected L-cells [17,48,55]. Interestingly, the data presented here are in line with a recent report on pancreatic β-cells, in which strong cell-cell-adhesion was correlated with low Rac1-activity along with IQGAP1-β-catenin association. Whereas enhanced motility was associated with high Rac1 activity and IQGAP1-Rac1 interaction [55]. In β-cells menin might be an important regulator in the differential interaction of IQGAP1 with Rac1 or the E-cadherin-adhesion complex [55]. These results point towards a cell type-specific regulation of Rac1-E-cadherin signalling and function depending on the availability of different IQGAP-binding protein, such as menin. Additional studies are necessary to explain these contradictory data about the role of Rac1 activity in cell-cell adhesion.

Based on data presented in this study as well as in several published reports [40,55,56], we would suggest the following model for the role of Rac1-IQGAP in the regulation of adherens junctions of epithelial pancreatic cells. Initial local activation of Rac1, as initiated by ligation of E-cadherin molecules located on opposing cells, is important for adherens junction formation. But further on, Rac1 activity disturbs the development of mechanically stable adherens junctions. IQGAP1 seems to play a key role in this context, although the molecular details are still puzzling. IQGAP1 binds Rac1, especially activated Rac1, as well as β-catenin. Our data suggest an association of IQGAP1/β-catenin with the E-cadherin complex which correlates with enhanced amounts of E-cadherin adhesion complexes associated with the actin cytoskeleton in epithelial cells of pancreatic origin. The association of IQGAP1 with active Rac1 results in destabilisation of adherens junction as a cause of enhanced E-cadherin protein degradation.

## Conclusion

In summary our data suggest that in pancreatic carcinoma cells activated Rac1 interacts with IQGAP1, which is associated with a disassembly of E-cadherin-mediated adherens junctions and with increased cell migration and invasion of pancreatic carcinoma cells. On the contrary, prolonged reduction of Rac1 activity contributes to a stabilisation of E-cadherin and E-cadherin/catenin-complexes followed by increased cell-cell adhesion. This stabilisation correlates with increased binding of IQGAP1 to the E-cadherin/catenin-complex. Our results emphasise a regulatory role of Rac1 in promoting pancreatic tumour cell migration and metastasis.

## Competing interests

The authors declare that they have no competing interests.

## Authors' contributions

BH, KM and IB performed the experiments and analysed the data. KG generated the cell lines and KG and AM designed the study, analysed the data and wrote the manuscript. All authors read and approved the final manuscript.
